# New Gold Nanostructures for Sensor Applications: A Review

**DOI:** 10.3390/ma7075169

**Published:** 2014-07-17

**Authors:** Yuanchao Zhang, Wendy Chu, Alireza Dibaji Foroushani, Hongbin Wang, Da Li, Jingquan Liu, Colin J. Barrow, Xin Wang, Wenrong Yang

**Affiliations:** 1 College of Chemistry and Chemical Engineering, Ocean University of China, Qingdao 266100, China; 2 School of Life and Environmental Sciences, Deakin University, Deakin, VIC 3217, Australia; E-Mails: yuanchao.zhang@deakin.edu.au (Y.Z.); wychu@deakin.edu.au (W.C.); alirezadibaji84@gmail.com (A.D.F.); d.li@deakin.edu.au (D.L.); colin.barrow@deakin.edu.au (C.J.B.); 3 School of Chemistry and Biotechnology, Yunnan Minzu University, Kunming 650031, China; E-Mail: wanghongbin@ynni.edu.cn; 4 College of Chemical Science and Engineering, Laboratory of Fiber Materials and Modern Textile, the Growing Base for State Key Laboratory, Qingdao University, Qingdao 266071, China; E-Mail: jliu@qdu.edu.cn

**Keywords:** gold nanowires (GNWs), gold nanoparticles (GNPs), nanoelectrodes, surface enhanced Raman spectroscopy (SERS), surface enhanced optical spectroscopy

## Abstract

Gold based structures such as nanoparticles (NPs) and nanowires (NWs) have widely been used as building blocks for sensing devices in chemistry and biochemistry fields because of their unusual optical, electrical and mechanical properties. This article gives a detailed review of the new properties and fabrication methods for gold nanostructures, especially gold nanowires (GNWs), and recent developments for their use in optical and electrochemical sensing tools, such as surface enhanced Raman spectroscopy (SERS).

## 1. Uncommon Gold: Old Material with New Applications

Gold based nanostructures are important research subjects in nanotechnology. The tremendous research and advanced application of gold nanostructures have emerged only in the recent decades. Actually, they are old materials used by ancient Chinese and Egyptians in the fifth or fourth century B.C. [1]. There are also evidences showing that ancient Romans used gold colloids to stain glass red or mauve. However, the first scientific literature of gold nanoparticles (GNPS) was reported by Michael Faraday in the 1850s [2]. The profound properties of GNPs have been gradually observed as important nanostructured materials. The highly favorable properties, including the large surface to volume ratio, unique optical and electronic properties, and easy surface modification, have brought intensive focus on GNPs from both research and industry. Many efforts have been devoted to tailor the properties of GNPs for specific applications, especially in sensor development. The morphology, solubility, surface functionality and stability of GNPs can be controlled via different synthetic routes, such as Turkevich method, Brust method, Perrault method and many other newly created approaches [1,2,3,4,5,6].

Another interesting gold nanostructure is gold nanowire. GNWs are one-dimensional nanostructures with high aspect ratio (L/D > 500) [7]. Because of the high surface to volume ratio, large anisotropy and self-assembly ability, GNWs have been used as building blocks for nanostructured sensing devices in chemistry and biochemistry fields [8]. GNWs can be divided into two types, single crystalline and polycrystalline. The single crystalline GNWs usually grow lengthwise via oriented attachment of crystalline GNPs with the diameter of single crystalline GNWs smaller than 2 nm, mostly depending upon the size of the nanoparticles [9,10,11,12]. The polycrystalline GNWs, with larger diameter (>5 nm), are also synthesized by assembly of stable atomic clusters, but they have shown different properties with single crystalline GNWs [13]. For example, polycrystalline nanowires exhibit excellent mechanical strength [14], but their electron transport properties are not as good as single crystalline GNWs, as their grain boundaries result in lower conductivity [8,15].

GNWs and GNPs have unique electrochemical, optical and magnetic properties which are different from their bulk [16]. Furthermore, the synthesis of GNWs and GNPs are greatly developed with more specific characteristics required for gold nanostructured biosensor devices.

## 2. Why Use Gold Nanostructures in Sensors?

### 2.1. Unique Properties of Gold Nanowires

GNWs can be used as important building blocks for nanotechnology because of their excellent physiochemical properties. As for unusual gold nanostructures, 70% of the gold atoms of ultrathin GNWs are at the surface, which make them an excellent nanoelectrode candidate in electrochemical applications, such as pressure sensors, DNA detector, interconnects and nanoelectrodes [8].

**Figure 1:**
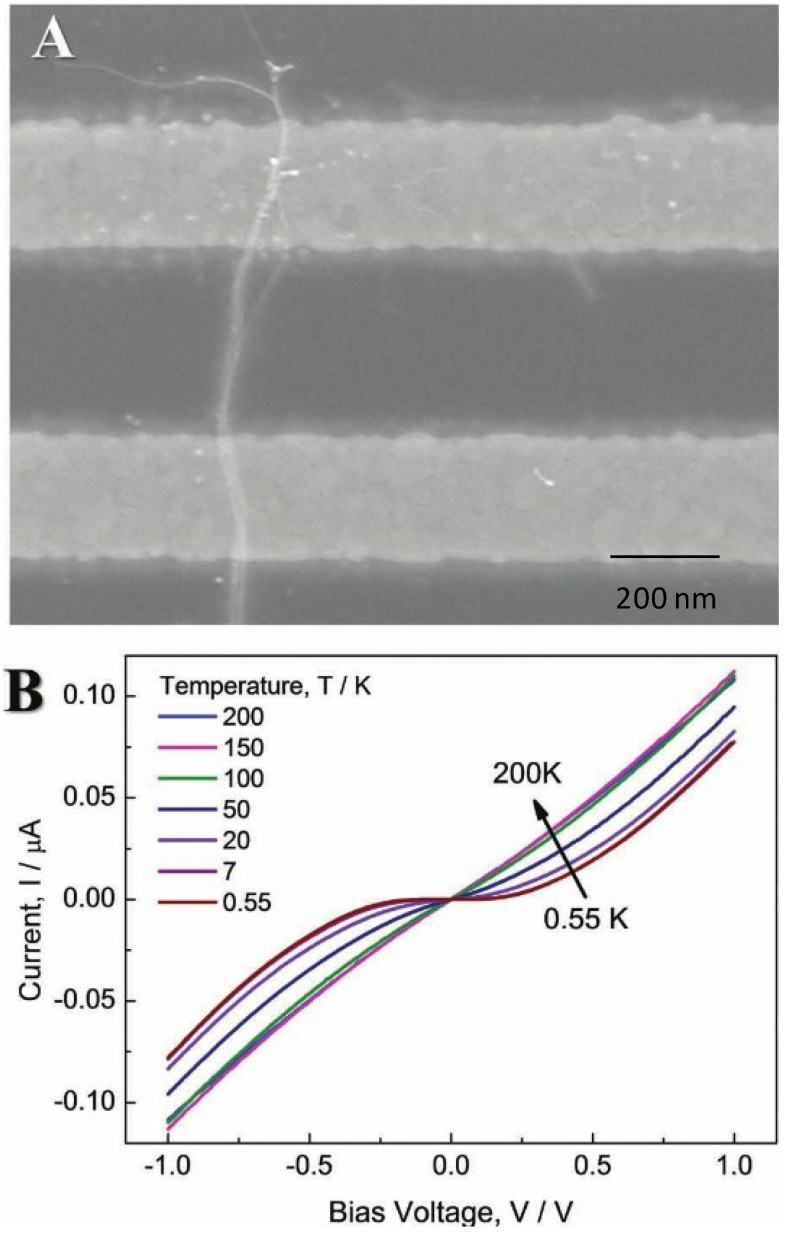
(**A**) SEM image of a rope of Au nanowires crossing an array of Au electrodes; (**B**) current *versus* voltage behavior of Au NW in different ranges of temperatures. Reprinted with permission from [15]. Copyright 2012, WILEY-VCH Verlag GmbH & Co. KGaA.

Meanwhile, GNWs could provide high current densities, high signal to noise ratio and low double layer capacitance. These properties are important for sensing applications [17]. As the GNWs are not thermally stable, mild heating could make them disintegrate into nanoparticles. Therefore, GNWs become stable with the help of an organic molecule layer, such as oleylamine (OA). However, as shown in Figure 1, the organic layer greatly deteriorated the electron transport of GNWs, whose resistance increases from 10^3^ Ω to 10^6^ Ω [15]. Catalytic ability can further be reduced by the organic layer [18]. So, removal of the organic layers is essential for practical applications. For example, the use of low concentration of sodium borohydride (NaBH_4_) solution removed the molecular adsorbates on GNWs immediately [19]. Thus, the pure GNWs without protective layers have fantastic conductivity and catalytic ability, and they can be greatly involved in sensing and catalytic fields.

The understanding of fracture behaviors and corresponding mechanisms is essential for ultrathin gold nanowires’ potential in nanoscale electrical and mechanical devices [20]. It not only helps in design and fabrication of next generation interconnects, but also advances our understanding of nanoscale mechanics and coupling of electrical and mechanical properties [14,21]. The stability of GNWs pressure sensors is greatly dependent upon the fracture behavior of GNWs. With the greatly developed cold welding techniques for ultrathin gold nanowires, the picking-up and clamping procedures are realized without damaging the sample structures and morphologies which make the systematic study of tensile fracture behavior for sub-20 nm gold nanowires available. As gold is considered one of the most ductile metals, the ductile fracture of GNWs are observed with plastic deformation and a neck formed in the middle section before final fracture. However, the unexpected brittle-like fracture of GNWs are reported which are closely related to the twin structures of GNWs. Furthermore, the brittle fractures have higher engineering strength (0.929 GPa) than ductile fractures. The mechanism of brittle-like fractures is related to the misalignment of twin structures formed during the initial loading stage, which is different with the plastic deformation for the ductile fracture [14].

Furthermore, gold nanostructures with surface plasmons resonance have enabled surface enhanced techniques essential for trace level detection, such as surface enhanced Raman spectroscopy (SERS) and surface enhanced fluorescence (SEF) [22]. GNWs, as cylindrical nanostructures, have attracted great attention owing to the tunable longitudinal localized surface plasmon resonances (LSPRs), whose plasmon absorption band redshift with the increasing aspect ratio (length/diameter) [23]. LSPRs are the collective coherent oscillations of electrons in metallic nanostructures. The enhanced optical near-field due to plasmon excitation in GNWs is of great interest in both basic research and for applications as chemical sensors and optical spectroscopy [4,24,25]. Moreover, GNWs can easily self-assemble into a two-dimensional network structure with a variety of closely packed nanowires which serve as an active substrate in SERS [4]. As for SERS, both local field and radiative enhancement contribute to the SERS cross-section. Maximum SERS enhancement is induced at an intermediate wavelength between peak excitation wavelength and Raman vibrational frequency which results in simultaneous enhancement of incident and scattered photons [26]. While in SEF, the fluorescence cross-section on gold surface depends on the relative enhancements of radiative and non-radiative decay rates through amplifying the local photonic mode density and synchronous energy transfer to the metal. Therefore, quenching is induced when fluorophore is close to the gold surface, while Raman signal is significantly enhanced. As distance increases, fluorescence intensity reaches a maximum and then falls off while Raman signal decreases greatly.

**Figure 2:**
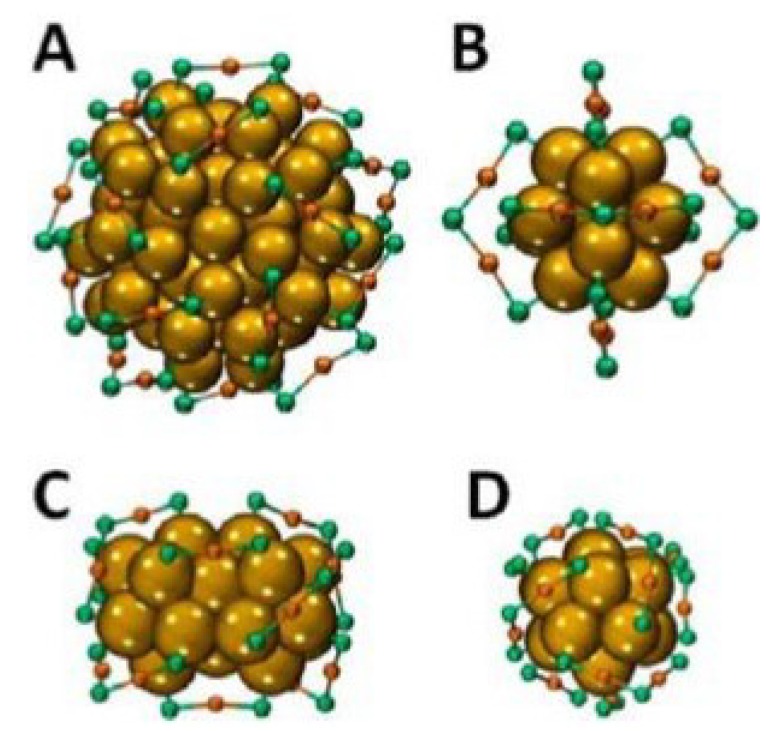
Chirality and crystal structures of the Au_102_ (p-MBA)_44_ cluster (p-MBA: p-mercaptobenzoic acid) (**A**); Au_25_ (2-PET)_18_ cluster (2-PET: 2-phenylethylthiolate) (**B**); and Au_38_ (2-PET)_24_ in side view (**C**) and along its principal axis (**D**). For the latter, the left-handed A-enantiomer is shown. The organic groups are removed. Yellow, Au_core_; orange, Au_Adatom_; green, sulfur. Reprinted with permission from [27]. Copyright 2013, American Chemical Society.

Last but not least, the chirality of gold clusters is important for the development of asymmetric drugs, sensors and catalysts. The chirality of gold clusters are considered in four levels, which includes the chiral thiolate ligands, the arrangement of the ligands, the inherent chirality of cluster core and the cis/trans isomerism in the bridged Au-S binding motif. The crystal structure of Au_102_ (p-MBA)_44_ (Figure 2A) is thought of as a 49-atom Marks decahedron and additional 30 surface atoms split into two 15-atom subgroups on opposite sites. Fivefold symmetry is found for the core of the cluster, in which 19 short and two long units SR (AuSR)_x_ (x = 1, 2) protect the core. The crystal structure of Au_25_ (2-PET)_18_ consists of a Au_13_ core with six dimeric units (Figure 2B) and the structure of Au_38_ (2-PET)_24_ consists of a face-fused bi-icosahedral Au_23_ core which is protected by six dimeric and three monomeric units (Figure 2C,D). The bridged binding motifs SR-Au-SR adopts cis/trans geometry with other organic ligands. For the diametric units SR (AuSR)_2_, the pseudochiral is considered for the central sulphur atom. With the development of high performance liquid chromatography (HPLC) and circular dichroism spectroscopy, the intrinsically chiral gold clusters are developed greatly for enantioseparation [27]. Furthermore, the Au_40_ (2-PET)_24_ clusters have Au_26_ core, which is composed of two icosahedrons in contact. The intrinsically chiral ligands are inverted from the enantiomers at high temperature and ligand exchange reaction involves chiral ligands to increase optical activity. Furthermore, the chirality of gold clusters is used in catalysis and sensing fields because of the flexibility of the Au-thiolate interface [28]. Table 1 gives a list of properties of gold nanowires and their potential applications.

**Table 1:** List of properties of gold nanowires and their potential applications.

Properties of GNWs	Potential application	Reference
Large surface area	Nanoelectrode Sensor, catalytic,	[8]
Electron transport with organic OA layer	High resistivity, 10^6^ Ω, temperature dependent non-linear behavior, lower catalytic ability	[15,17]
Electron transport without OA layer	Low resistivity, 10^3^ Ω, high electron transport, electrochemical, nanoelectrode sensor, high catalytic ability	[15,17]
Mechanical properties	High engineering strength, interconnects	[14,20]
LSPR	SEF, SERS	[21,22,23,24]
Chirality of gold cluster	Asymmetric drugs, sensors and catalysts	[27]
Easy self-assemble	Active substrates for SERS	[4]

### 2.2. Unique Properties of Gold Nanoparticles with Surface Modification

GNPs, with a diameter between 1 nm and 100 nm, have been extensively used in chemical and biological sensors because of their excellent physical and chemical properties. The unique optical property of GNPs is one of the reasons that GNPs attract tremendous interests from different fields of science, especially in the development of sensors. The spherical GNP solutions show a range of vibrant colors including red, purple and violet when the particle size increases, and they could be used to stain glass in ancient time. The intense color is caused by the strong absorption and scattering of *ca.* 520 nm light [29], which is the result of the collective oscillation of conduction electrons on the surface of GNPs when they are excited by the incident light. This phenomenon is known as surface plasmon resonance (SPR), and it depends greatly on particle size and shape. Thus, the SPR peak is tunable by manipulating the size of GNPs, and this property cannot be observed on bulk gold and GNPs with a diameter smaller than 2 nm. The SPR peak is not only sensitive to size and the shape, but also many factors such as protective ligand, refractive index of solvent, and temperature. The interparticle distance particularly shows great influence on SPR. Therefore, the red-shifting and the broadening of the peak are observed when GNPs are aggregated due to analyte binding. The color change of aggregated GNPs from red to blue is the principle of colorimetric sensing. A number of recent researches and reviews provide a detailed discussion on the factors that influence the SPR of GNPs [6,30,31,32,33]. The emitting of the SPR can be used for measuring electrical properties. When metal nanoparticles form 1D, 2D or 3D structures, the combining of surface plasmons from the neighbor nanoparticles results in new optical properties which depend on the extent of assembly. According to the work by Creighton *et al.* [34], different metal ions have different SPR band positions in the UV/Vis spectrophotometer.

Apart from absorption and scattering properties, GNPs also display fluorescence properties which could be utilized in sensor fabrication. Photoluminescence can be generated from GNPs under certain conditions, such as laser [35] and ultraviolet (UV) excitation [36]. GNPs can enhance or quench the fluorescence of fluorophore depending on the distance between the fluorophore and the particle [37]. Based on radiating plasmon model, Lakowicz suggested the absorption and scattering are related to the enhancing or quenching the fluorescence. As incident energy is dissipated by absorption and far-field radiation is created by scattering, larger GNPs are more likely to enhance fluorescence. Smaller GNPs are more likely to quench fluorescence because the scattering component is dominant over absorption [38]. Fluorescence is enhanced because the far-field radiation from the fluorophore is reflected back on itself. Hence, fluorescence can be quenched by fluorescence resonance energy transfer (FRET) [39], photoinduced electron transfer (PET) [40] or nanosurface energy transfer (NSET) [41] pathway.

In addition to the fluorescence property, GNPs can enhance Raman scattering, which leads to the development of SERS based sensing. Raman scattering is normally weak. The electromagnetic contribution to SERS is induced by the intense optical frequency field which originates from the plasmon resonance of GNPs, which can be used for single molecule detection [42]. The enhancement of Raman scattering depends on various factors including particle size, shape and aggregation. There are now two mechanisms to illustrate how SERS is enhanced, one is long-range electromagnetic effect and the other is short range chemical effect. The electromagnetic contribution to SERS is induced by the intense optical frequency field which originates from the plasmon resonances of GNPs, while the chemical mechanism is induced by the electron transfer between molecules and the surface. From electromagnetic, the enhancement factor is about 10^11^, which can only be obtained at the interstitial sites between two particles or at outside sharp surface protrusions. As for single molecular detection, electromagnetic enhancement is not strongly enough, there should be chemical or other related enhancement to accomplish single molecular detection [43].

Please note that GNPs alone have limited application in sensing unless surface modification is performed. Careful selection and design of ligands strongly influence the sensitivity and selectivity of a sensor. Thiol-gold chemistry is well studied and has been widely used for the surface coating of GNPs. The strong S-Au bond (*ca.* 50 kcal·mol^−1^) [44] allows effortless anchoring of a range of chemical species, regardless of their molecular sizes, to the surface of GNPs [1,45,46]. The hybrid materials, containing inorganic metal core and organic layer, show novel properties and can self-assemble into the desired structure, which could be essential for biosensing. Van der Waals’ force, electrostatic interaction and solvation force are important forces for tuning GNPs’ assembly [47,48,49,50]. However, the immobilization of chemical species also affects the stability of GNPs, as it changes the properties of the solid–liquid interface. In this way, GNPs may aggregate and the functionality may not be maintained. This could be solved by the addition of surfactant or stabilizer [51], or the optimization of ligand and GNPs. Table 2 summarizes potential applications based on some of the properties of GNPs.

**Table 2:** List of properties of GNPs and their potential applications.

Properties of GNPs	Potential applications	Reference
SPR	Colorimetric, SERS based sensing	[6,30,31,32,33]
Photoluminescence Fluorescence enhancement Fluorescence quenching	Fluorescence sensing	[30,31,32,33,35]
Raman scattering enhancement	SERS by electromagnetic and chemical mechanism	[37]
Strong light scattering	Dynamic light scattering (DLS) assay	[38]
Easy surface modification	Molecular recognition in different sensing systems	[1,45,46,47,48,49,50]

### 2.3. Principles of LSPR

The interaction of light with nanostructures is the focal domain of nanooptics. The general optical properties of metal nanostructures are significantly different from their bulk material because of the quantum size effect which is from discrete electron bands. When plane-wave lights excite a GNP, coherent oscillation of conduction electrons are induced by the oscillating electric field which leads to the accumulation of polarization charges on the surface of a GNP, known as LSPR [26,52,53,54,55]. The electric field of light interacts with the free electrons in the nanoparticles, leading to a charge separation and in turn the Coulomb repulsion among the free electrons acting as a restoring force pushes the free electrons to move in the opposite direction. Mie theory involves Maxwell’s equation to describe the LSPR for non-interacting GNPs, with the extinction cross-section, C_ext_, which can be expressed in Equation (1) as:

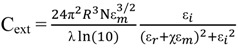
(1)
where ε*_m_* is the dielectric constant of the surrounding medium; ε*_r_* and ε*_i_* are the real and imaginary part of the dielectric function of particles; *R* is the radius of the GNP; and N is the electron density. The factor χ stands for the shape of the particle, and is assigned a magnitude of two for a spherical particle and can be 20 for particles with high aspect radio.

As for interacting particles, the plasmon resonance red-shifts and a lower energy absorption band appears, resulting from the interactions between GNPs: near-field coupling and far-field dipolar interactions. Furthermore, surface plasmons for non-spherical metallic nanostructures are shape-dependent. In particular, for GNWs the plasmon resonance splits into low- and high-energy absorption bands, one for the transverse mode around 515 nm and the other for the longitudinal mode whose position depends strongly on the aspect ratio [46,56,57]. In the case of gold nanorods, Gans theory describes the optical behavior according to Gan’s formula, the extinction cross-section C_ext_ in Equation (2) as:

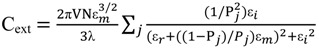
(2)
where V is the volume of the particle; and P*_j_* is the depolarization factor. The depolarization factor for the elongated particles can be described as:

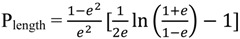
(3)

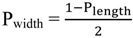
(4)
where *e* is the ellipticity as:

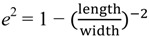
(5)


The LSPR occurs when ε*_r_* = −((1 − P*_j_*)/P*_j_*) ε*_m_*, where P*_j_* = P_longth_ for the longitudinal plasmon resonance and P*_j_* = P_width_ for the transverse plasmon resonance. Small changes in the aspect ratio will change the plasmon band significantly.

## 3. Synthesis of Gold Nanostructures

### 3.1. Synthesis of Gold Nanowires

GNWs are normally prepared by two methods, “bottom-up” and “top-down” methods. Bottom-up method is the self-assemble of small sized structures to form larger structures, while the “top-down” method is based on the reduction of large systems into smaller sizes [58]. Bottom-up methods are more popular which include template directed methods and non-template directed methods.

#### 3.1.1. Template Directed Methods

The template directed synthesis process can be divided into two types: soft template and hard template. During the process of crystal growth of GNWs, anisotropy is controlled by the asymmetry of templates, such as mesoporous materials, nanocrystals, and so on [59].

For a soft-template method, GNWs growth process is constrained by chemical or electrochemical reduction, depending on structure-directing molecules whose role is to inhibit the growth of certain crystal faces while improve the growth of certain faces. For example, Oleylamine (OA) is now commonly used as both growth template and reducing agent, which reacts with AuCl (or HAuCl_4_) to fabricate GNWs. The mechanism of this process is based on the formation of an [(Oleylamine) AuCl]_n_ inorganic polymer, which works as a template for the formation of ultrathin nanowires with uniform thickness, uniform diameter of approximately 1.6 nm and length up to 4 μm [60]. Another explanation of the growth mechanism is oriented attachment of gold nanoparticles, from which two facets fuse together to form a single particle. Ascorbic acid is added into AuCl and OA solution to remove the amine from the gold surface, which leads the GNWs grow lengthwise (111) [61]. Since this method usually takes several days to complete, a more rapid synthesis method appears by adding triisopropylsilane (TIPS) as a highly effective reducing agent which shortens the reaction time to 5 h [4].

The distance for the self-assembly of GNWs is controlled in a closely packed parallel array. Ultrathin GNWs with different distances are prepared by using different organic molecules with different alkyl chain lengths (abbreviated as C_n_AA where n is 12, 14, 16 or 18), which tunes the space of the parallel array. The average gap distance are 2.13, 2.29 and 2.65 nm for C_12_AA, C_14_AA, C_16_AA, and C_18_AA [62].

Surface properties of GNWs are further controlled by self-assembly of amphiphilic molecules on their surface, which changes the dispersion ability of GNWs in solution. For example, the hydrophobic surface of GNWs makes them collective at the air–water interface in an aqueous solution with the presence of cetyltrimethylammonium bromide (CTAB), an amphiphilic molecule as both a coordination ligand and a capping agent [13,63].

As for a hard-template method, porous membranes are used as a rigid template for chemical and electrochemical growth. Unidirectional 1-D vertical aligned arrays of nanowires are produced in this way. In the fabrication process of GNW arrays with hard templates, such as track-etched polycarbonate and anodic aluminum oxide (AAO), single crystals GNWs form at a given current density in the electrochemical method. The nucleation rate is proportional to the cross-sectional area of the nanowires. Single crystal GNW arrays are fabricated when the pore diameter is less than 70 nm for AAO template [64].

Moreover, graphene oxide (GO) can be used as template to fabricate GNWs because of the abundant oxygen functional groups (–OH, –COOH, epoxy). The advantage of GO template is well distributed GNWs growth on GO rather than bundles as the functional groups of GO direct the growth of GNWs. Different crystal phases of GNWs, hexagonal Close Packed (hcp) and face centered cubic (fcc), are observed to both grow on the surface of GO [65].

#### 3.1.2. Non-Template Directed Methods

The most commonly used non-template methods involve electron beam lithography, electrodeposition, and many other newly developed methods [66]. GNWs with defined geometry are fabricated by electron beam lithography through selective removal of either the exposed or non-exposed regions [67,68,69,70]. With the development of electrodeposition, lithographically patterned nanowire electrodeposition (LPNE) can produce polycrystalline GNWs through a sacrificial nickel nanoband electrode and recess a horizontal trench into a defined thickness of GNWs [58].

Apart from the above introduced methods, more newly developed methods are attracting scientist’s attention for the preparation of nanowires. Nanoskiving is a new method for fabricating complex nanostructures with defined geometry. By depositing gold onto an epoxy substrate, slicing the section and then etching the epoxy resin, GNWs are fabricated [17]. This nanoskiving method is confirmed to be a fast method for GNWs’ preparation. Table 3 gives a list of synthesis methods of gold nanowires.

**Table 3:** List of synthesis methods of gold nanowires.

Synthesis of GNWs	Advantages	Disadvantages	Diameter (nm)	Length (μm)	Reference
Soft Template OA	easy to make	longer time-several days, organic layer	1–2	3–5	[60]
OA and ascorbic acid	easy to make	longer time several days organic layer	1–2	3–5	[61]
OA and TIPs	fast-several hours	hard control, organic layer	1–2	3–5	[4]
Soft template C_n_AA	tune gap distance	C_n_AA dispersion, organic layer	1–2	3–5	[62]
Amphiphilic molecules CTAB	growth in water	organic layer	1–2	2–3	[63]
Hard template AAO	control diameter	etch the template	2–100	1–10	[64]
Hard template GO	well dispersed not bundles	hard to separate	1–2	3–5	[65]
Electron beam lithography	control morphology	expensive	10	1–10	[56,57,58]
LPNE	define thickness	polycrystalline	10	1–10	[58]
Nanoskiving	fast and low lost	need special instrument	10	1–10	[17]

### 3.2. Synthesis of Gold Nanoparticles

Since Faraday synthesized colloidal gold in 1857 [2], many efforts have been made to prepare GNPs in different sizes, shapes, solubility, and surface functionality, in order to fulfill the demands from various scientific fields. In most cases, GNPs are synthesized in solution phase by reducing gold salts in the presence of an appropriate capping agent that prevents particle aggregation.

Reducing tetrachloroauric acid (HAuCl_4_) in water by sodium citrate, invented by Turkevich *et al.* [71] in 1951, has been the most popular method. This protocol typically prepares spherical monodisperse GNPs of 10–20 nm, while sodium citrate acts as both a reducing and capping agent. Frens later refined this method to synthesize GNPs of 16 nm to 147 nm by manipulating the ratio of HAuCl_4_ to sodium citrate [72]. The problem of poor size and shape dispersion in large citrate-reduced GNPs (>50 nm) was solved by Perrault’s group [73]. Better quality of GNPs of 50–200 nm can be obtained using hydroquinone (HQ) as a reducing agent.

Aside from aqueous phase, GNPs can also be prepared in organic phase. In 1994, Brust *et al.* [74] introduced a breakthrough two-phase synthetic method to prepare 1–3 nm GNPs. HAuCl_4_ is transferred into toluene using tetraoctylammonium bromide (TOAB) and reduced by sodium borohydride (NaBH_4_) in the presence of alkanethiol. Hostetler *et al.* [75] extended the range of size of GNPs to 5.2 nm by carefully controlling the reaction temperature, the ratio of HAuCl_4_ to alkanethiol, and the reduction rate. Other capping agents, such as phosphine [76] and TOAB [77], have been used to synthesize GNPs of diverse sizes and dispersities.

In addition to the bottom-up methods mentioned above, top-down methods, including laser ablation [78], are another way to obtain GNPs. The size and shape of prepared GNPs can be modified by many treatments, such as digestive ripening [79] and laser irradiation [78]. According to Table 4, the GNPs are generally stabilized with chemicals of low functionality, which limits their applications in sensing. Versatile molecules, such as protein and oligonucleotide, can be anchored on GNPs by ligand exchange process for the detection of a variety of analytes of interest. Summarized in Table 4.

**Table 4:** List of synthesis methods of gold nanoparticles.

Size (nm)	Synthetic method	Capping agent	Reference
1–2	Reduction of HAuCl_4_ by NaBH_4_ in toluene	Phosphine	[76]
1–5.2	Reduction of HAuCl_4_ by NaBH_4_ in toluene	Alkanethiol	[74,75]
3–5	Reduction of HAuCl_4_ by NaBH_4_ in toluene	TOAB	[77]
10–147	Reduction of HAuCl_4_ by citrate in water	Citrate	[71,72]
50–200	Reduction of HAuCl_4_ by HQ in water	Citrate	[73]

## 4. Recent Development in the Use of Gold Nanoparticles Based Materials in Sensors

### 4.1. Gold Nanoparticles Based Colorimetric, Fluorescence and SERS Sensing

For human healthcare and environmental monitoring, GNP based sensors have been extensively developed to detect metal ions. The strong interaction between metal ions and glutathione (GSH) is a common design concept for metal sensing. Beqa *et al.* [80] have reported a colorimetric and DLS assay for lead (II) ion (Pb^2+^) using GSH functionalized GNPs at pH 8. Aggregation of GNPs, induced by the chelation of Pb^2+^ via carboxylate groups, is detected by DLS with a detection limit of 100 ppt (0.4 nM). Other than river water sample, this sensing system has also successfully detected Pb^2+^ in paint and plastic toys, which extends its application. Pentapeptide (CALNN) has been used to stabilize GSH coated GNPs to detect Pb^2+^ in both aqueous solution and living cells [81]. High sensitivity (2.9 × 10^−15^ M Pb^2+^ per cell) has been achieved.

**Figure 3:**
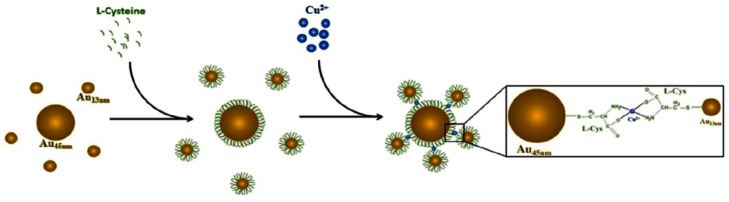
Schematic representation of the protocol used to synthesis Cu^2+^ mediated core-satellite structure by using gold nanoparticles (GNPs) of two different sizes. Reprinted from reference [82] Copyright 2013, with permission from Elsevier.

Apart from functionalizing uniform size GNPs, Weng *et al.* [82] has demonstrated the linkage of large and small L-cysteine coated GNPs as core-satellite by copper (II) ions (Cu^2+^) as shown in Figure 3. The self-assembly of core-satellite structure causes red shift of SPR peak and allows detection of Cu^2+^ in aqueous solution as low as 2.23 μM. Further, Cu^2+^ in living cells can be detected by a bovine serum albumin (BSA) coated GNPs fluorescent sensor [83]. The addition of Cu^2+^ binds with BSA and quenches the fluorescence of GNPs, which can be reversed by adding glycine. The limit of detection (LOD) was reported to be 50 μM. Another fluorescent sensor is fabricated to detect mercury (II) ions (Hg^2+^) by DNA functionalized GNPs [84]. The design is based on the interaction of thymine and Hg^2+^, which reduces the distance between the fluorescein and GNP and results in fluorescence quenching. GNPs coated with Raman reporter dye and cadmium (II) ion (Cd^2+^) sensitive polymers have been used as a SERS based sensor [85]. The presence of Cd^2+^ induces aggregation and activates the dye with 90-fold signal enhancement. Although the LOD is 1 μM, which is relatively low among SERS based sensors. This system allows the detection of Cd^2+^ in heavily colored samples.

However, relatively few researches have been carried out to develop GNP based sensors for anions. It is mainly due to the difficulty in designing receptors for anion. He *et al.* [86] have developed a 4′-(4-mercaptophenyl)-2,2′,6′,2′′-terpyridine zinc(II) complex (MPTP-Zn) coated GNPs colorimetric sensor for phosphate ion (PO_4_^3^^−^) in aqueous solution. The design is based on binding of PO_4_^3^^−^ to Zn metal complex in enzymatic reaction, rather than relying on the conventional hydrogen bonding and electrostatic reaction. Although the detection is rapid and highly selective, the LOD is reported to be 120 μM. Therefore, the relatively low sensitivity restricted the practical application of this system. Unlike common sensing approach, a colorimetric sensor for sulphide ion (S^2^^−^) by GSH functionalized GNPs is designed to make use of the anion-for-molecule ligand exchange reaction instead of the functionality of GNPs [87]. The partial replacement of GSH by S^2^^−^ caused aggregation with LOD of 5 μM.

Fluorescein isothiocyanate (FITC) labeled BSA has been coated on GNPs to detect both iodide (I^−^) and cyanide ion (CN^−^) in high salinity solution [88]. FITC is released and its fluorescence is restored during the binding of anion to GNPs. The LOD was 50 nM and 1 μM for I and CN, respectively, in the presence of various masking agents. CN^−^ is a popular analyte of interest. Lou’s group also contributed to the development of fluorescence sensing of CN^−^ based on the dissolution of GNPs [89,90]. Imidazole functionalized polyfluorene and polyacetylene were attached on GNPs, and LOD was reported to be 0.3 μM and 3 μM, respectively. SERS based sensing for CN^−^ has been developed considering the formation of hot spots during the aggregation of ascorbic acid functionalized GNPs in the presence of CN^−^ [91]. High sensitivity (110 ppt) is achieved with excellent discrimination against other metal ions and anions.

GNPs based sensors have been developed to detect biomolecules as well. Ligation chain reaction (LCR) based GNP aggregation is widely utilized in the design of DNA sensors. Yin’s group and Shen’s group have reported colorimetric LCR based systems with LOD of 0.1 × 10^−18^ M by DLS and 2 × 10^−17^ M by UV-Visible spectrometer, respectively [92,93]. GNP probes were ligated during hybridization of target strand, and the aggregation caused detectable color change after a number of thermal cycles.

**Figure 4:**
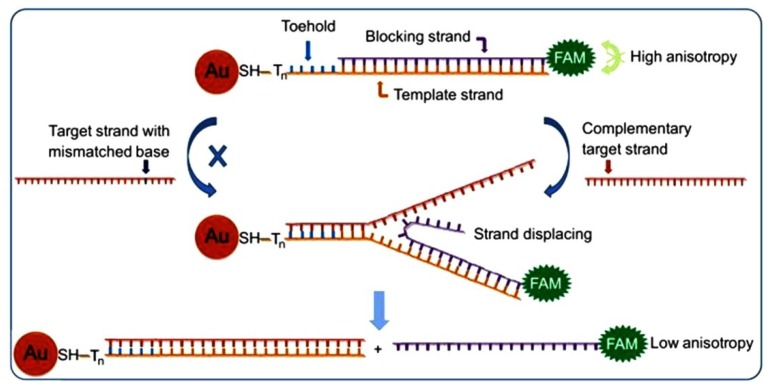
Schematic representation of toehold-mediated stand-displacement reaction based GNP enhanced fluorescence anisotropy for single nucleotide polymorphism (SNP) detection. Reprinted from reference [94], Copyright 2013, with permission from Elsevier.

As shown in Figure 4, Wang *et al.* [94] have explored the usage of toehold-mediated stand-displacement reaction with GNP fluorescence anisotropy signal enhancement in the development of a homogeneous single nucleotide polymorphism (SNP) detection assay. Fluorescein labeled DNA is detached during the hybridization of target DNA, resulting in low fluorescence anisotropy. The dynamic and kinetic difference allows distinguishing SNP from perfectly matched target strand. Florescent sensing of oligonucleotides can also be carried out by modifying GNPs with hairpin shaped molecular beacon (MB), which the fluorescence is quenched without the presence of target analyte. Xue *et al.* [95] have developed such a sensor for mRNA of signal transducer and activator of transcription 5B (STAT5B) that is related to the metastasis and proliferation of tumor cells. The conformation of MB is changed when hybridized with target, thus fluorescence is enhanced due to the increase in fluorophore-GNP distance. Similarly, bi-MB functionalized GNPs sensor detecting of two types of breast cancer related mRNA is reported [96]. Target analytes from 0.3 to 0.5 nM are stated as the LOD of this assay. Both systems successfully show fluorescence in living cells which are promising in early detection of cancer.

Some research contributes to the detection of smaller biomolecules. A colorimetric sensor for GSH has been developed based on the anti-aggregation of GNPs [97]. It has been reported that higher selectivity could be achieved by the use of anti-aggregation of GNPs, and the LOD is 8 nM. Plasmodium falciparum heat shock protein (PfHsp70), antigen for malaria parasite, is detected by a fluorescence competitive immunoassay using antibody coated GNPs [98]. Free antigen displaced the fluorophore labeled recombinant PfHsp70, thus fluorescence enhancement was observed. The LOD is estimated to be 2.4 μg·mL^−1^ and this assay has detected antigen in human blood culture at a 3% parasitemia level. GNP-based sensors can also detect biological pathways. The monitoring of DNA assembly and enzyme cleavage is achieved by SERS based sensor [99]. DNA assembly creates hot spots for detection, while enzyme cleavage decreases the SERS signal. Thus, the whole process can be monitored by observing the changes in SERS signals.

The sensitivity of GNP-based sensors is influenced by various factors, including the analyte of interest, protective ligand and sensing approach. Some GNP-based sensors utilizing their optical properties are summarized in Table 5.

**Table 5:** Comparison of various GNP-based sensors.

Sensing approach	Analyte	LOD	Reference
Colorimetric	Metal ion (Pb^2+^)	0.4 nM	[80]
	Anion (S^2−^)	5 μM	[87]
	Oligonucleotide (ssDNA)	0.1 aM	[92]
	Biomolecule (GSH)	8 nM	[97]
Fluorescence	Metal ion (Hg^2+^)	16 nM	[84]
	Anion (CN^−^)	0.3 μM	[89]
	Oligonucleotide (mRNA)	0.3 nM & 0.5 nM	[96]
	Biomolecule (PfHsp70)	2.4 μg·mL^−1^	[98]
SERS	Metal ion (Cd^2+^)	1 μM	[85]
	Anion (CN^−^)	4 nM	[91]

### 4.2. Gold Nanowires Based Sensing Applications

#### 4.2.1. Gold Nanowires Based Electrochemical Sensing Applications

**Figure 5:**
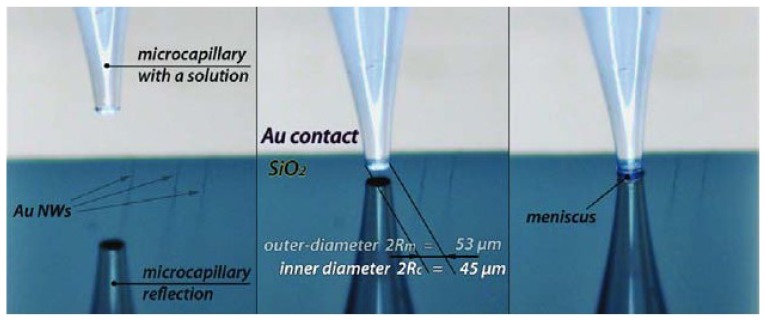
Process of microcapillary positioning over GNWs and the contact of the solution meniscus with the NW electrode. Reprinted with permission from [100]. Copyright 2011, American Chemical Society.

One dimensional GNWs have now been greatly developed in nanoelectronics because of their high aspect ratio, unusual physical properties and potential applications, such as pressure sensors, DNA detector, interconnects and nanoelectrodes [9]. As for nanoscale electrodes (NSEs), people have devoted great effort to fabricate all kinds of NSEs, such as disk or conical electrodes based on tapered or etched metal wires, in the past two decades. With the development of the lithographic processing technique, NSEs have been greatly developed in the sensing field (Table 6). However, there are drawbacks for this method including inherent variability of electrode-insulator seal and contamination. Recently, a simple photolithography-free fabrication of GNWs’ electrode was developed by gold electrodeposition onto ultra-long, flow-aligned, single-walled carbon nanotubes (SWNTs) (Figure 5). The electro-transfer (ET) kinetic quantification is studied with a microcapillary-based electrochemical method. Through this method, the trace level fluid of interest (confined by the microcapillary) is precisely located on the GNWs by moving the capillary [100]. From the LSVs’ study, ET kinetic of GNWs is studied, with K^0^ = 0.10 cm·s^−1^.

**Table 6:** List of GNWs based electrochemical sensing applications.

GNWs based materials	Advantages		Properties and application		Reference	
GNWs NSEs	Microcapillary based electrochemical method and lithographic-free electrodeposition method		ET kinetics of GNWs K^0^ = 0.10 cm·s^−1^		[100]	
GNWs with tunable electron transport	Tunable electron transport		Crossover from a non-Fermin Liquid TLL ground state to a disordered state with VRH layer		[101]	
Giant superlattice nanomembrane	Mechanical strong, optical transparent		Thickness 2.5 nm, resistance is 1142 kΩ		[102]	
Pressure sensor with GNWs	Low energy consumption High sensitivity >1.14 kPa^−1^ Fast response time <17 ms High stability >50,000		Pressure force reduce the wire to wire spacing		[103]
Removal the layer of GNPs	Rapid removal using NaBH_4_ solution in water		Hydride has a higher binding affinity to gold than organothiols		[19]
DNA template GNWs sensor	Circle amplification of single strand DNA		DNA detection of limit LOD is 6.6 × 10^−15^ M		[104]
Lattice orientation protection in gold nanowires by a zipper	Ag blocks preserving the lattice of gold rings		Zipper mechanism shows ligand loss, lattice alignment and coalescence.		[105]

With a growing need for interconnects or circuit elements, GNWs are potential candidates for next generation interconnects because of their good conductivity and high aspect ratio. The Coulombic interaction and random background disorder from extrinsic and intrinsic factors influence the charge transport ability of 1-D nanowires. Ultrathin GNWs grow on SiO_2_/Si and Si_3_N_4_substrates which are separated via organic layers with different thickness (6 nm, 2 nm and 0 nm). Through chemical adhesion of nanowire to the substrates using a structured link layer, the influence of extrinsic and intrinsic disorder sites on the transport are controlled which shows a clear crossover from a non-Fermin Tomonaga-Luttinger Liquid (TLL) ground state to a disordered state with variable range hopping (VRH) mediated transport. The TLL is established via clear power-laws, whereas a VRH governed by electron–electron interactions define the localized regime. The linker layer can lead to different effects, such as the electronegative groups lowering the electron density of the nanowires [101].

GNWs have also been developed greatly and used as building blocks because of their stability and self-assemble ability. Recently, mechanically strong, optically transparent, giant superlattice nanomembranes are fabricated from ultrathin GNWs, with 2.5 nm thickness and tens of micrometres in length. The square resistance of membrane is about 1142 kΩ, which is higher than model predicted sheet resistance of 15–200 Ω because of the large contact resistance from the organic layer at the joint of two nanowires by preventing electron hopping from wire to wire. However, with a pressure force to the multilayer membranes, the conductivity increased by 15%–19% which is due to the decrease in wire to wire spacing [102].

**Figure 6:**
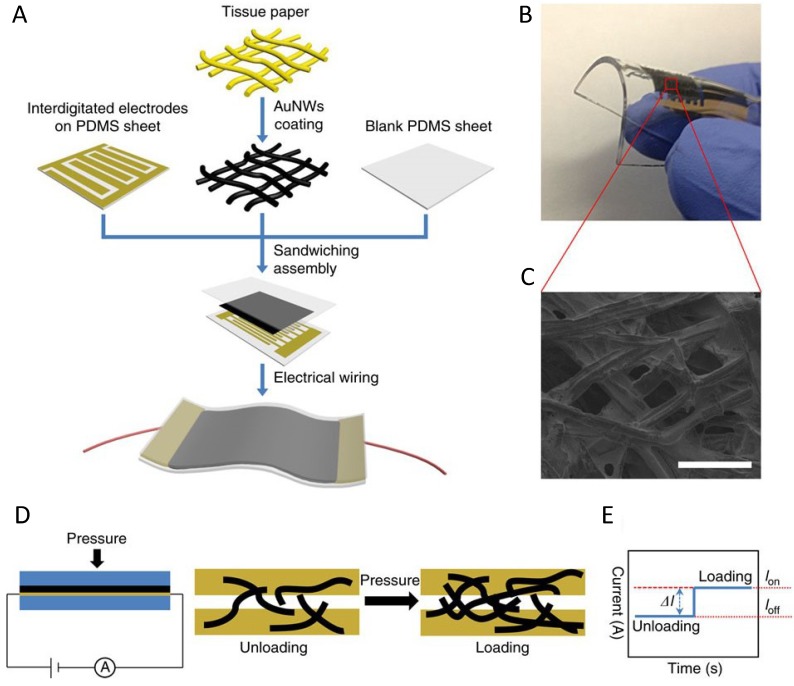
Pressure sensor based on the GNWs coated tissue paper. (**A**) Schematic illustration of the fabrication of a flexible pressure sensor; (**B**) photograph showing the bendability of the sensor; (**C**) scanning electron microscopy image of the morphology of gold nanowires coated tissue fibers (scale bar, 100 μm); (**D**) schematic illustration of the sensing mechanism; (**E**) current changes in responses to loading and unloading (*l*_off_: unloading; *l*_on_: loading). Reprinted by permission from Macmillan Publishers Ltd.: [Nature Communications] [103], Copyright 2014.

GNWs are further used for pressure sensors owing to the sensitivity of pressure force. The pressure sensors are usually designed in principle of the changes in capacitance, piezoelectricity and resistivity facing outside pressures. GNWs-impregnated tissue paper pressure sensors (Figure 6) are prepared using polydimethylsiloxane (PDMS) sheet and a patterned PDMS sheet with inter-digitated electrode arrays. Apart from pressure force, other types of mechanical forces, *in-situ* artery wrist pulses and acoustic vibrations can also be detected. Large area integration and patterning can also be obtained by current mapping of pressure distribution [103].

Surface modification of GNPs is used to improve functionality, biocompatibility, and target specificities. However, the organic layer is found to be detrimental to electronic transport and catalytic ability. The removal of the organic layer through a convenient method has attracted many researchers’ interests for a long time. The use of NaBH_4_ in water can remove molecular adsorbates rapidly and completely, such as organothiols, thiophene, adenine, rhodamine, small anions and polymer (poly(*N*-vinylpyrrolidone)). It is not clear whether this method would break the nanowire structures [19].

**Figure 7:**
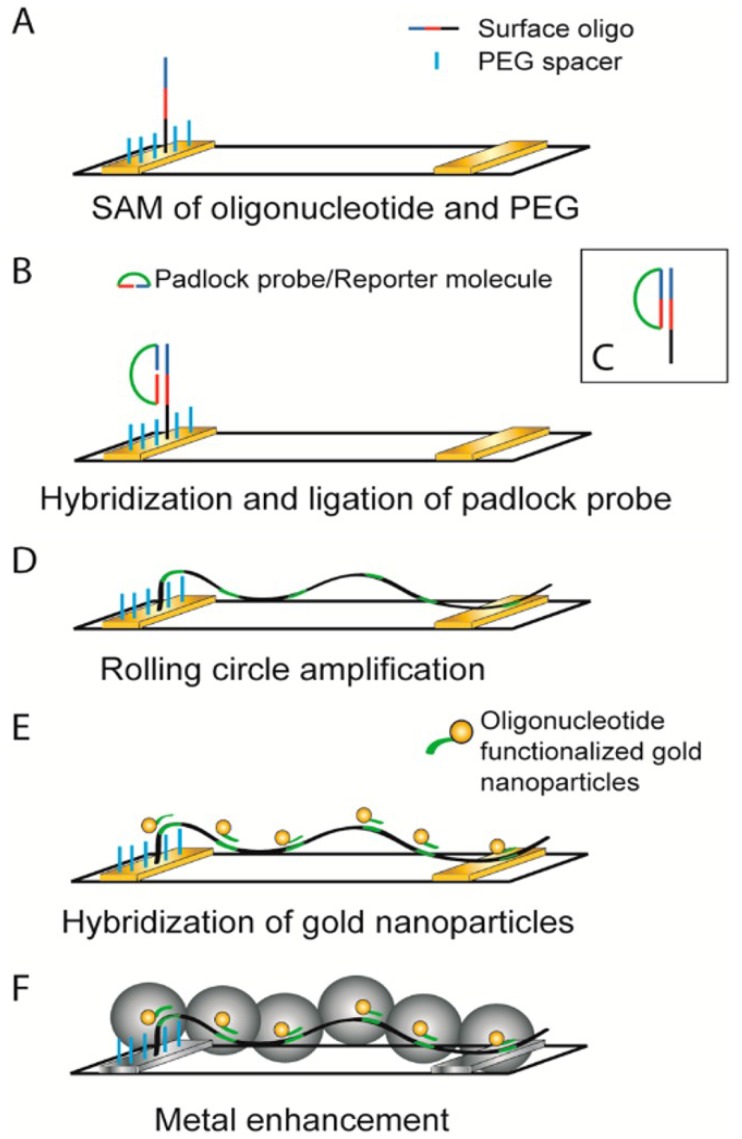
Schematic illustration of formation of metalized wires from stretched rolling circle amplification products to generate an electrical signal. A padlock probe, whose ends have been designed to hybridize head-to-tail on a target sequence, is hybridized and ligated to a self-assembled monolayer (SAM) of oligonucleotides containing the target sequence and poly(ethylene glycol) (PEG) alkane backfiller on a gold electrode (**A**–**C**); (**D**) This circle is then used for RCA, creating a long single-stranded product on the electrode; (**E**) Oligonucleotide-functionalized gold nanoparticles are then hybridized to the product; (**F**) With the aid of these particles, the single-stranded DNA threads are metalized using either a silver or gold salt solution to form metal wires. Reprinted with permission from [104]. Copyright 2014, American Chemical Society.

Furthermore, GNWs can be fabricated through self-assemble of GNPs along DNA molecules. DNA has unique molecular self-assembled properties as molecular glue. However, the poor electrical conductance limits its applications in the sensing field. This problem is solved by coating metal on the DNA surface, such as silver, gold, and palladium. The resistance of DNA decreases from 10^12^ to 5 Ω. Moreover, the DNA based sensors are greatly developed for point of care (POC) diagnostics. As shown in Figure 7, DNA template GNWs based electrode is fabricated though GNPs seed-mediated growth of GNWs along the DNA template. Additionally, using the circle amplification of single stranded DNA with repetitive sequences in highly selective recognition reactions, nucleic acid and proximity ligation detection are accomplished. Through the linear relationship between the amount of *E. coli* DNA and counted blobs, the LOD is 6.6 × 10^−15^ M and the signal to noise ratio reaches nearly 10 orders of magnitude [104].

In addition, GNWs can be coalesced into gold rings which still keep the axial orientation of the original GNWs. After the low temperature coalescence, Ag blocks are grown to preserve the Au lattice. The ring’s diameter is about 476 ± 85 nm and GNW bundle width is 27 ± 8 nm. The mechanism for reconciling the interlocked events is explained by a zipper mechanism which includes ligand loss, lattice alignment and coalescence [105].

#### 4.2.2. Gold Nanowires Based Optical and SERS Sensing Applications

##### 4.2.2.1. Gold Nanowires Based Optical Sensing Applications

GNWs exhibit interesting optical properties with numerous applications in sensing, such as their surface plasmon resonances (Table 7). They are used to confine electromagnetic radiation to a volume of sub wavelength dimensions. For example, vibrational spectroscopy of molecules can be obtained by surface enhanced scattering technique, such as SERS and surface enhanced IR absorption. As for IR adsorption, the plasmon field of the GNW resonance produces an exceptional, high intensity enhancement at the tip ends with resonant interaction of vibration dipoles with a broadband IR plasmonic resonance [106,107]. With individual GNW, vibration signal can be enhanced by up to 5 × 10^5^ for molecular monolayers absorbed on GNWs. The increasing enhancement is achieved using nanogaps between GNWs. GNWs offer great opportunities as waveguides, because of deep sub-wavelength confinement, coherence maintenance and low scattering losses [108]. Moreover, surface enhanced optical diffraction [24] and fluorescence also show great potential for chemical and biochemical sensors [24].

**Table 7:** List of the flowing resent researches on optical properties of GNWs sensing.

Materials	Methods and Properties	Results (Factor)	Reference
GNWs arrays	Optical Diffraction methods	Used for surface molecule adsorption process	[24]
GNWs arrays with DNA	ssDNA hybridizing, optical diffraction measurements	Detect sequence of unlabeled ssDNA	[109]
GNWs with different cross section	Scattering loss and joule heating with cross section	Scattering loss dependent on plasmon mode rather than cross section	[110]
Gold nanoantenna dimmers	Infrared spectroscopy	Bonding and antibonding combination show	[111]
Bowtie gold nanoantenna	Surface enhance fluorescence	Factor of 1340	[112]

GNWs have attracted much attention because of their tunable cylindrical longitude LSPRs. Red shift occurs with an increase of aspect ratio (length/diameter). GNWs’ wavelength-tunable, surface plasmon-based, resonant-sensing application is expected to enhance the sensitivity of evanescent-wave in chemical and gas sensors. GNWs arrays have surface plasmon-enhanced diffraction at optical wavelengths due to a transverse LSPR adsorption. Furthermore, the maximum diffraction intensity pattern of GNW arrays 115 nm in width is 633 nm [24]. Optical diffraction methods play quite an important role in the detection of molecular adsorption processes.

**Figure 8:**
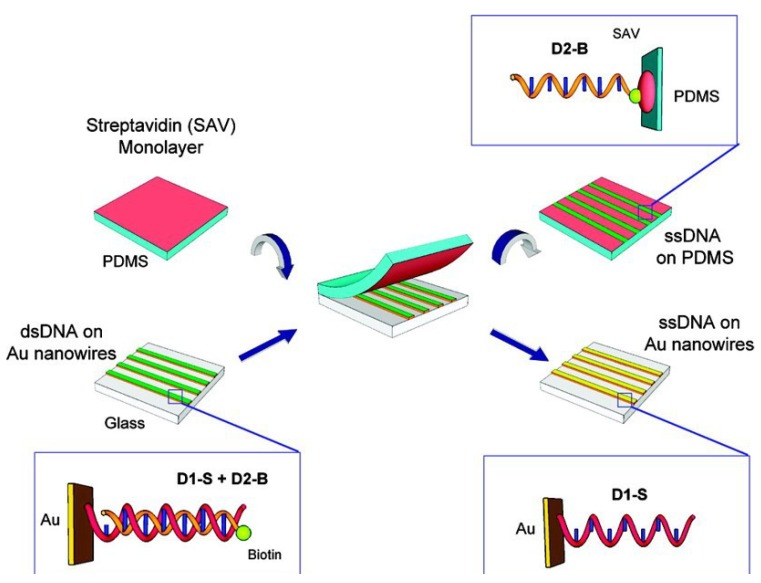
Schematic diagram of the master-replica transfer process for nanoscale single strand DNA patterns on PDMS polymer substrates. The GNWs array master was “linked” by first exposing it to thiol-modified ssDNA, D1-S and then hybridizing with complementary biotinylated ssDNA, D2-B, to create a dsDNA monolayer on the nanowire surface (**bottom left**). A streptavidin-modified PDMS substrate (**top left**) was placed in contact with the linked GNWs array with minimum pressure. When peeled apart, D2-B was bound to PDMS replica by the strong biotin–streptavidin interaction (**top right**), leaving D1-S on the nanowire master (**bottom right**). DNA sequence of D1-S is 5′-HS-(CH_2_)_6_-TTT TTT TTT TTT TTT TTT TTT TTT TTT TTT TTT TTT TTT TTT TTT-3′ and D2-B is 5′-biotin-(CH_2_)_6_-AAA AAA AAA AAA AAA AAA AAA AAA AAA AAA-3′. Reprinted with permission from [109]. Copyright 2010, American Chemical Society.

As shown in Figure 8, GNWs can be used in DNA detection, which are linked with biotinylated single strand DNA by hybridization adsorption. Double-stranded DNA can be formed between the transferred single strand DNA nanowires and single strand DNA in solution, which is used to detect a specific target sequence of unlabeled single strand DNA [109].

**Figure 9:**
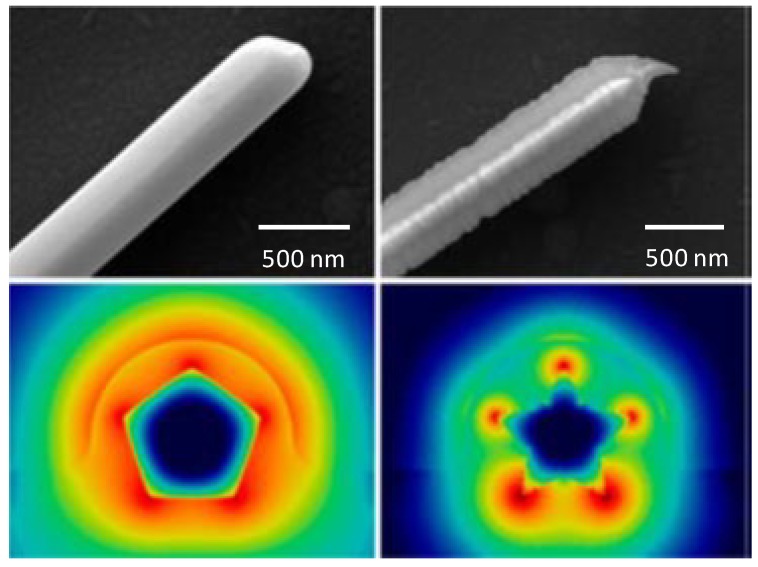
GNWs with pentagonal section and five-point star cross section. Reprinted with permission from [110]. Copyright 2014, American Chemical Society.

One-dimensional metallic wires are of great interest in nanoscale optical devices because of the ability to confine light to sub-wavelength dimensions by surface plasmon polaritons (SPPs). However, the metallic nanowires suffer significant SPP propagation losses at optical frequencies through scattering and joule heating which shorten the SPP propagation length to a few tens of micrometers. While chemically prepared GNWs have a high degree of crystallinity and smooth surfaces, their scattering losses of optical and SPP waveguiding properties are reduced compared to plasmonic structures fabricated by metal evaporation, leading to longer SPP propagation lengths. The propagation length is related to not only the diameter of the nanowires, but also to the cross section shape. As shown in Figure 9, Nauert *et al.* [110] investigated the pentagonal and five pointed star cross section of GNWs. They addressed how the cross section and SPP mode number influenced the absorptive and radiative losses. GNWs with a five-point star cross section have a shorter propagation length and a higher coupling efficiency than those with a pentagonal cross section. This is because the electric fields are located at the sharp ridges of GNWs, which leads to higher absorptive losses [110], while scattering losses were found to be dependent on the plasmon mode rather than cross sectional geometry.

Gold nano-antenna dimmers resonant in the infrared spectral were studied. The results indicate that the decreasing of the gap to below 4 nm results in the increasing plasmonic coupling and a red shift of the surface plasmon resonances to lower energy. Both the bonding and antibonding combination of the individual antennas are emerged in the IR transmittance, which resulted from both structural defects and small gaps [111].

Sensitive plasmonic materials enable detection of biological analytes, such as protein biomarkers, DNA, or enzyme. In the experiment of bowtie gold nanoantenna coated with *N*,*N*’-bis(2,6-diisopropylphenyl)-1,6,11,16-tetra-[4-(1,1,3,3-tetramethylbutyl)phenoxy]quaterrylene-3,4:13,14-bis(dicaboximide) TPQDI molecules in thin poly(methyl methacrylate) (PMMA) layer, the single molecule’s fluorescence is enhanced by up to a factor of 1340, which is a result of greatly enhanced absorption and an increase in the radiative emission rate, leading to enhancement of the intrinsic quantum efficiency [112,113]. Native low quantum efficiency emitters have higher fluorescence brightness enhancements because the intrinsic quantum efficiency can be improved by the presence of the antenna.

##### 4.2.2.2. Gold Nanowires Based SERS Sensing

Surface enhanced Raman scattering (SERS) has received great interest due to various attributes, such as excellent selectivity, and rapid detection capability [4] (Table 8). The commonly used SERS substrate depends on nanogaps as the enhancing units. At these hot spots, excitation and Raman scattering light can couple with the surface plasmons and be electromagnetically enhanced. For example, the intensity of Raman enhancement from a single gold sphere (60 nm) to two gold spheres with 5 nm gap can be increased from 24 to 1686 times. Furthermore, the resonance would redshift from 522 to 550 nm by coupling between the spheres. Apart from larger Raman dipole moments, the more directional radiated fields also results in larger SERS electromagnetic enhancement. The importance of directionality in SERS is studied, which is essential for obtaining very high EFs (average value up to 10^10^). SERS is a powerful method for single molecule detection because of a larger electro field enhancement factor [43].

**Table 8:** List of the recent studies on SERS.

SERS substrate materials	Detection of molecules	Enhanced factors	Reference
Gold sphere with 5 nm gap single molecular detection	Directional radiated fields	EFs average value up to 10^10^	[43]
DNA origami NPs two with 3.3 nm gap	Far field scattering	A small number of dye molecules	[114]
GNWs based DNA	Raman carrier Cy5	Strong SERS signal	[115]
DNA template GNPs	SERS	EFs up to 10^6^	[116]
Single nanowire based sensors of Hg^2+^	Hg^2+^	Detection limit up to 1 × 10^−10^ M	[117]
Ag-Au bimetal nanocages	–	SERS enhanced intensity	[118]
Au-Cu alloy nanotube	4-Mpy as a single molecule	SERS enhanced intensity	[119]
Dimers antennas	Energy momentum spectroscope/radiated power	Perpendicular to the dipole orientation	[120]

**Figure 10:**
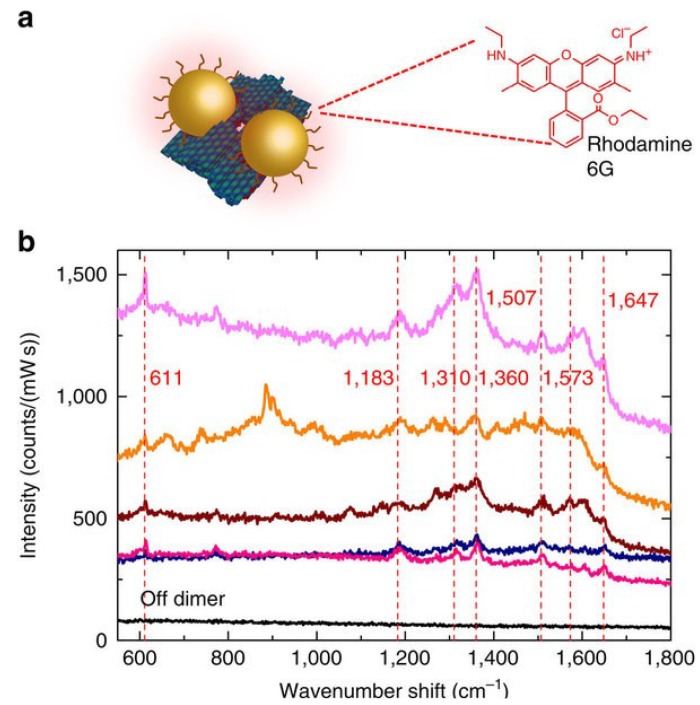
SERS measurements of an external analyte. (**a**) SEBS measurements of a thin layer of Rhodamine 6G adsorbed onto individual dimer structures plated on a gold-coated silicon wafer; (**b**) each spectrum peak is clearly visible (indicated by a dashed line). By contrast, a spectrum taken from a region away from the dimers (“off dimers”) is of low intensity and does not display any peaks. Reprinted by permission from Macmillan Publishers Ltd.: [Nature Communications] [114], Copyright 2014.

DNA origami-assemble nanoparticle dimers also provide single molecule spectroscopic techniques such as surface enhanced fluorescence and SERS (Figure 10). As for SERS, a much larger enhancement factor is needed for single molecule detection because the cross-sections of SERS are 10 orders of magnitude smaller than fluorescence. Individual gold particles are positioned by self-assembly, through origami technique. The individual dimers are fabricated with two 40 nm GNPs with gaps of 3.3 nm. Plasmonic resonance peaks significant red shift through far field scattering measurement because of strong plasmonic coupling [114]. A small number of dye molecules and single stranded DNA oligonucleotides are detected by this DNA origami assemble SERS active NPs.

**Figure 11:**
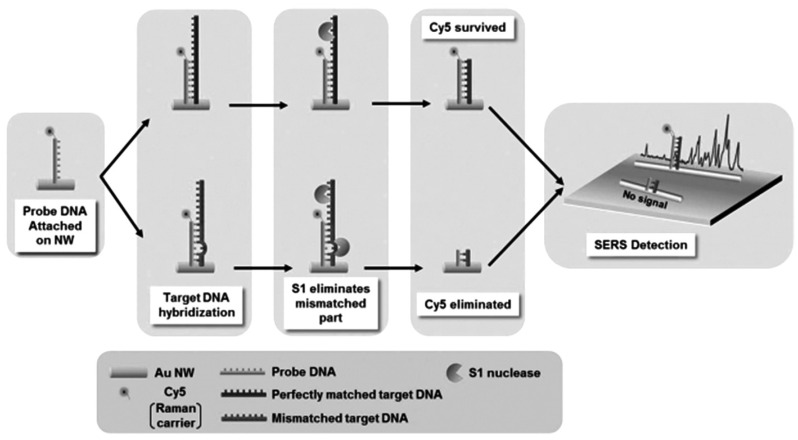
Schematic representation of the strategy for the detection of single-base-mismatched sequences in DNA. Reprinted with permission from [115]. Copyright 2014, WILEY-VCH Verlag GmbH & Co. KGaA.

GNWs can easily self-assemble into 2-D structure with nanogaps (less than 1 nm) which make GNWs based substrate attractive for SERS. For example, single nucleotide polymorphisms (SNPs), as biomarker for genetic disease, can be detected for the early diagnosis. The DNA probe is first attached to the GNW through a thiol group via the strong Au-S bond. When perfectly matched target DNA is hybridized with a DNA probe, it protects the DNA probe from S1 nuclease attack. Then, the Raman carrier Cy5 retains its function to provide a strong SERS signal as shown in Figure 11 [115].

**Figure 12:**
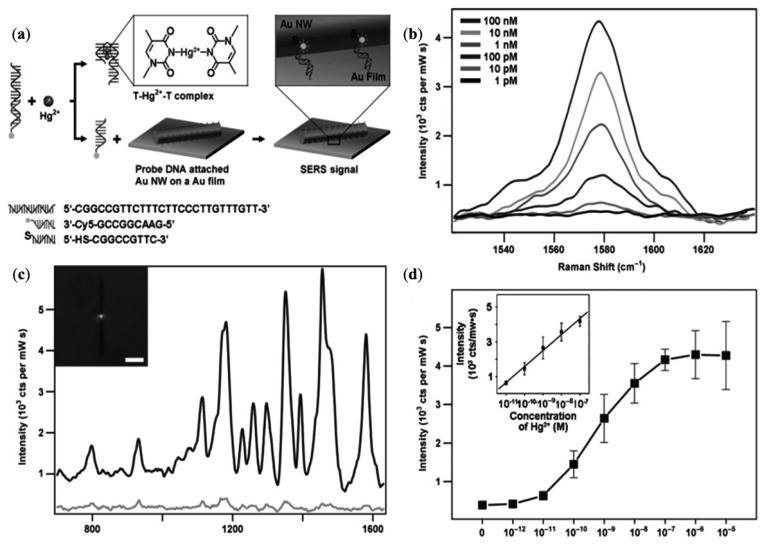
Schematic representation of a single nanowire-on-film (SNOF) SERS sensor for Hg^2+^ detection based on a structure-switching dsDNAs (**a**); SERS spectra of a SNOF sensor in the absence of (**c**,**d** spectrum) and addition of 1 μm Hg^2+^ solution (**a**,**b** spectrum). The inset is an optical image of a SNOF sensor and the scale bar denotes 5 μm (**c**); 1580 cm^−1^ band intensities of Cy5 from SNOF sensors by varying the concentration of Hg^2+^ (**b**); Plot of 1580 cm^−1^ band intensities *versus* concentrations of Hg^2+^. The inset shows a dynamic range and linearly fitted line. The date was obtained from five measurements and the error bars represent standard deviation (**d**). Reprinted with permission from [117]. Copyright 2011, WILEY-VCH Verlag GmbH & Co. KGaA.

DNA-template GNPs self-assemble into nanowires for enhanced SERS and catalytic applications. DNA-GNWs were prepared by exposing the DNA-Au salt solution under UV light, while the diameter and length of the nanowires could be tuned by controlling the various reaction parameters [116]. The self-assembled structures of DNA-GNWs generate a highly stable and reproducible SERS signal with enhancement factor up to 10^6^. As shown in Figure 12, single GNW on film SERS sensor can also be used as an ultrasensitive and selective sensor by the use of structure switching to double stranded DNAs. The conformational changes of the double stranded DNAs will be induced. A Raman reporter will be released by binding Hg^2+^. Through this method, the detection limit can reach 1 × 10^−10^ M [117].

**Figure 13:**
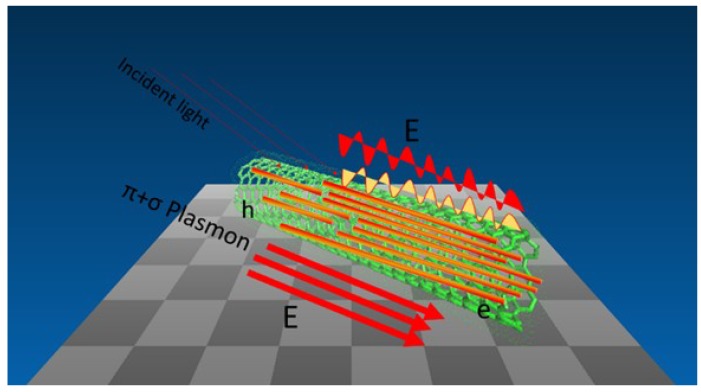
Scheme of hybrid GNWs/SWCNTs as SERS substrate for trace level detection [121].

One-dimensional (1D) nanomaterial exhibits excellent electronic and optical properties associated with its dimensionality and the quantum confinement effect and is used as building blocks for potential devices. In our group, we synthesize a new hybrid nanostructure SERS substrate with excellent enhanced ability using gold nanowires and single-walled carbon nanotubes for trace level detection (as shown in Figure 13). The one dimensional hybrid nanostructure serves as a multifunctional substrate for SERS, which shows higher enhancement properties. Additionally, after the removal of the oleylamine layer from the surface of the hybrid GNWs/SWCNTS, a higher Raman enhancement is achieved. Based on the new understanding through Raman with such a new nanomaterial, we provide a novel platform for trace level detection using the Raman enhancement [121].

The LSPRs have been of great interest because of their tunability to various aspects to modify the local field, such as the shape of the NPs. The composition of NPs is also important for catalysis and sensing applications and it is a useful method to control electronic properties of sensing applications. Through the study of the relationship between SERS intensities and composition of Ag-Au bimetallic nanocages, the wavelength of excitation plays a decisive role in determining the SERS capability of a bimetallic nanoparticle. As for the 514 nm excitation, with the increasing of Au ratio in the nanoparticle, the intensity of SERS is decreased, while for 785 nm excitation this trend is not observed suggesting the involvement of Au interband transition as a damping mechanism to attenuate the SERS intensity [118]. In addition, Cu-Au alloy nanotubes with five-fold twinned structures are prepared based on Cu nanowires as templates. The Au-Gu alloy shows a high enhancement of the SERS signal owing to the synergy effect between Cu-Au alloying, the galvanic replacement reaction and the nanoscale Kirkendall effect [43,119].

**Figure 14:**
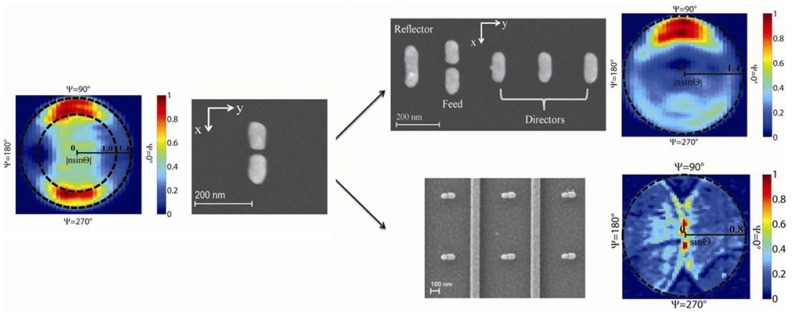
Raman emission pattern from YU antennas on dielectric substrate. SEM and measured emission pattern of thiophenol 1074 cm^−1^ Raman line retrieved from energy momentum spectroscopy measurements. Color map represents the scattering intensity normalized by the maximum intensity. Inner and outer black dashed circles indicate the critical angle at the air-glass interface and the numerical aperture of objective lens, respectively. Reprinted with permission from [120]. Copyright 2012, American Chemical Society.

Raman emission pattern from optical dimer antennas is studied with energy momentum spectroscopy. The majority of the radiated power goes into the substrate, perpendicular to the dipole orientation, and symmetrically into two lobes at the air-dielectric interface. As for the YU antennas, most of the Raman scattering occurs in the forward direction and the backward scattering is suppressed with F/B ratio to be 4.4 (Figure 14). In addition to the radiation emission pattern of device combing dimer antennas with plasmonic substrates, the Raman scattering is shaped into a narrow beam which could be efficiently collected by a low numerical aperture objective lens [120].

## 5. Conclusions and Future Perspectives

This paper gives a detailed review regarding the properties and fabrication methods for gold nanostructures—GNWs and GNWs—and their recent development in optical and electrochemical sensing applications, such as surface enhanced Raman spectroscopy. Through their excellent properties and great applications as discussed in this paper, gold nanomaterials have been significantly developed and have become more and more important in biochemistry and biotechnology. In recent years, GNWs (1-D nanostructures) have attracted researchers’ interest over other gold nanomaterials. One-dimensional nanomaterials have some advantages. Firstly, GNWs are easier to use for hybrids with other materials as building blocks than other gold nanomaterials because of their 1-D nanostructures with large surface area and physiochemical properties. Secondly, GNWs have more synthesis methods than GNPs which can be used in many specific applications.

Even with these tremendous advantages, GNWs also have some disadvantages, such as being easily influenced by the surrounding environment and breaking into nanoparticles. Also, GNWs are less conductive due to the outside organic layer. Furthermore, GNWs lose stability when removing the outer layer. Moreover, GNWs are flexible and are easily self-assembled into bundles in solution which might affect the ability of GNWs in biosensing applications.

GNWs nanostructures and most of their applications, such as nanoelectrodes and biosensors, are still in an early stage of development. As a result, deep understanding of GNWs is still needed. Some fundamental physical and chemical properties of GNWs have been simulated without being confirmed through experiments. The surface chemistry of GNWs must be deeply understood which determines mutual interaction with other nanomaterials. In future research work, more attention will be paid to the reproducibility and robustness of GNWs assemblies, due to their weaker thermal properties and less stable mechanical properties, which are closely related to the performance of applications. Scientists have invested great efforts in developing methods to synthesize robust GNWs and develop GNW sensors. There has recently been much progress in the preparation and performance of GNW nanostructures, but development in the reproducibility and robustness of GNWs shall never end.

## Nomenclature

NPs: Nanoparticles
NWs: Nanowires
GNPs: Gold nanoparticles
GNWs: Gold nanowires
SERS: Surface Enhanced Raman Spectroscopy
LSPR: Localized surface plasmon resonances
HPLC: High performance liquid chromatograph
SPR: Surface plasmon resonance
PET: Photoinduced electron transfer
FRET: Fluorescence resonance energy transfer
NSET: Nanosurface energy transfer
DLS: Dynamic light scattering
AAO: Anodic aluminium oxide
Hcp: Hexagonal Close Packed
Fcc: Face centred cubic
TOAB: Tetraoctylammonium bromide
